# Resolving Cell Population Heterogeneity: Real-Time PCR for Simultaneous Multiplexed Gene Detection in Multiple Single-Cell Samples

**DOI:** 10.1371/journal.pone.0006326

**Published:** 2009-07-27

**Authors:** Alan Diercks, Heather Kostner, Adrian Ozinsky

**Affiliations:** Institute for Systems Biology, Seattle, Washington, United States of America; Charité-Universitätsmedizin Berlin, Germany

## Abstract

Decoding the complexity of multicellular organisms requires analytical procedures to overcome the limitations of averaged measurements of cell populations, which obscure inherent cell-cell heterogeneity and restrict the ability to distinguish between the responses of individual cells within a sample. For example, defining the timing, magnitude and the coordination of cytokine responses in single cells is critical for understanding the development of effective immunity. While approaches to measure gene expression from single cells have been reported, the absolute performance of these techniques has been difficult to assess, which likely has limited their wider application. We describe a straightforward method for simultaneously measuring the expression of multiple genes in a multitude of single-cell samples using flow cytometry, parallel cDNA synthesis, and quantification by real-time PCR. We thoroughly assess the performance of the technique using mRNA and DNA standards and cell samples, and demonstrate a detection sensitivity of ∼30 mRNA molecules per cell, and a fractional error of 15%. Using this method, we expose unexpected heterogeneity in the expression of 5 immune-related genes in sets of single macrophages activated by different microbial stimuli. Further, our analyses reveal that the expression of one ‘pro-inflammatory’ cytokine is not predictive of the expression of another ‘pro-inflammatory’ cytokine within the same cell. These findings demonstrate that single-cell approaches are essential for studying coordinated gene expression in cell populations, and this generic and easy-to-use quantitative method is applicable in other areas in biology aimed at understanding the regulation of cellular responses.

## Introduction

The broad aim of much research is to decode the complexity of the human body, which is composed of at least 210 distinct eukaryotic cell types. The challenge is to determine which cells are responsible for particular biological activities, to identify the regulatory mechanisms and elements that control them, and to determine how pathology develops when those mechanisms go awry and cause disease. However, while the cell is recognized as a fundamental unit, only a limited number of measurement techniques permit single cell resolution. Standard techniques average the responses of cell populations and thus obscure inherent cell-cell heterogeneity and restrict the ability to distinguish between the individual responses of different cells within a sample[1], [2], [3], [4], [5], [6], [7], [8]. While these bulk techniques are useful for characterizing the spectrum of possible cellular responses, this approach severely compromises our ability to disentangle the complexity of the regulatory mechanisms controlling specific responses within a heterogeneous cell population.

Measurements with single-cell resolution are likely to greatly impact many areas of research, particularly the study of rare cells (such as immune cells active at the initiation of vaccination or cancer stem cells), and the analysis of samples of limited volume (such as human blood). For example, immune cells (such as macrophages and T cells) secrete numerous cytokines and chemokines to coordinate the regulation of defenses against infection, and to control immune activation during vaccination. Defining the timing, magnitude and the coordination of these cytokine responses will be critical to understanding the development of effective immunity. However, since the relevant responses occur within a subpopulation of cells, the responses of individual macrophages must be distinguished. Further, it is particularly desirable to measure the patterns of multiple cytokine responses from individual cells in order to decode the signaling pathways regulating these differential responses. While studies have achieved global analysis of one single-cell[9], [10], to gain insight into the behavior of a population, it is necessary to analyze multiple single-cell samples.

Cytokine measurements typically are performed by ELISA assays on cell populations, though a limited number of cytokines can be measured with single cell resolution by intracellular cytokine staining and flow cytometry. Using flow cytometry, single macrophages typically show more than 10-fold variation in their level of cytokine production, even in apparently uniform cell populations, such as cloned cell lines[11]. However, the flow cytometry approach to cytokine measurement is restricted by the paucity of affinity reagents capable of detecting cytokine protein expression in fixed/permeabilized cell samples and the limiting number of fluorescent channels available for multiplexing.

Multiplexed mRNA expression analysis with single-cell resolution is possible using fluorescence *in-situ* hybridization (FISH) and has the additional benefit of yielding histological information, but the degree of multiplexing is generally limited to 3-5 targets [12].

Recently, several instruments have been described that provide alternative formats to detect the expression of multiplexed genes in small samples. The devices from Fluidigm and Biotrove utilize microfluidics to position samples for high-throughput real-time PCR[13], [14]. These instruments are capable of processing 48–96 samples. Single samples also can be processed for real-time PCR on the Fluidigm instrument to count mRNA molecules[15], [16]. Nanostring also offers an instrument that uses direct detection to count mRNA molecules[17]. However, it is not yet clear how to use the digital counting approach on hundreds of samples in a single run. All three of these instruments provide solutions to the real-time PCR or detection step, but are not solutions to the cDNA synthesis step.

The ability to analyze multiple samples of single cells by integrating cDNA synthesis with the multiplexed mRNA expression analysis on each cell remains unrealized and is the goal that has inspired the research effort described in this paper. Standard mRNA purification and cDNA synthesis procedures used for cell populations (∼>10000 cells) involve affinity columns, and wash and precipitation steps that are not suited for the processing of samples derived from single cells. Therefore, we employed a methodology where cell lysis, mRNA purification and cDNA synthesis occurs in a single well, through the sequential addition of the necessary enzymes and buffers, without intervening cleanup steps, and especially without removing the sample from the well. In addition, we used flow cytometry to achieve absolute control of the number of cells in the sample.

We present data on the expression of 5 genes in each of 84 individual cells, with the sensitivity of at least 30 copies of mRNA and a fractional error of 15%. We utilize this method to characterize cytokine gene expression during macrophage activation. Remarkably, while cytokines appear coordinated in cell population assays (‘pro-inflammatory cytokines’ induced by highly similar cell signaling pathways), our single cell analyses reveal that the level of production of one cytokine in a cell is not predictive of the level of production of other cytokines within the same cell. These results have implications for efforts to define how inflammation is regulated, and the generic nature and scalability of the method make it applicable to many other areas in biology.

## Results

### Sensitivity to detect gene expression in single macrophage cells

**Figure 1:**
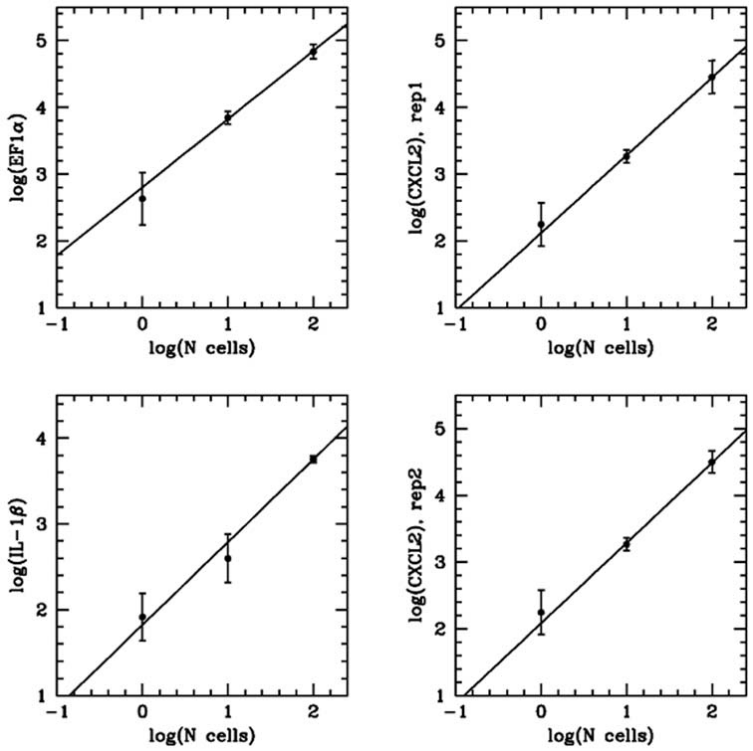
Sensitivity for single-cell mRNA measurements. Macrophages were activated with the bacterial stimulus, lipopolysaccharide (30 ng/ml for 2 hours), and the indicated number of cells (1, 10 or 100 cells) were sorted by flow cytometry. mRNA expression of the indicated genes was measured by real-time PCR using 1/8^th^ of the sample cDNA lysate per measurement. The mean and standard deviation of 12 samples are presented for each of the indicated number of cells. The C_t_ values were arbitrarily scaled to log_10_ values (y-axis).

We used the CellsDirect (Invitrogen) single-well procedure to perform cell lysis and reverse transcription to generate cDNA. Using flow cytometry, we sorted 1, 10, or 100 cells into individual wells of a 96-well microtiter plate containing 5 µL of lysis buffer. An aliquot (2 µL) of the cDNA synthesis reaction (16 µL) generated from each single-cell sample was taken forward for quantification by real-time PCR, which indicated that the mRNA for a variety of genes in single cell samples was easily measured at the expected levels, as determined by comparison to the gene expression levels measured in 10- and 100-cell samples (**Figure 1**).

**Figure 2:**
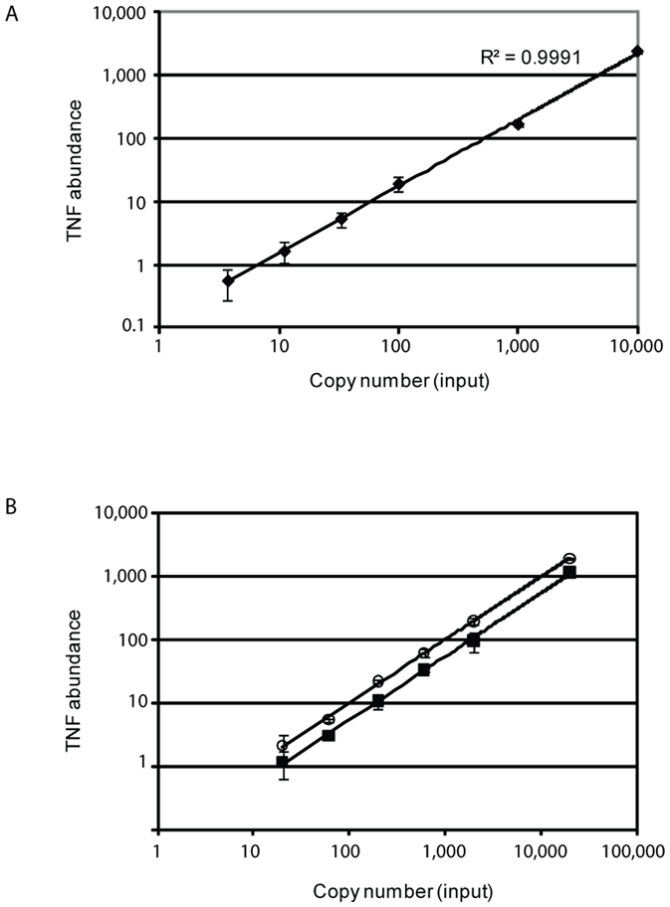
Sensitivity of detecting numbers of DNA and RNA molecules. Real-time PCR was performed over a concentration range from A) *TNF* DNA standard template or B) *TNF* mRNA standard template. cDNA synthesis was performed on the dilution series of mRNA samples and the difference in signal between the same amount of input mRNA (▪) versus input DNA (ο) indicates a reverse transcription efficiency of 54% in this experiment. For each copy number, the mean and standard deviation are shown for 12 samples in A) and 4 samples in B). The Y-axis is a log_10_ rescaling of the C_t_ values.

We examined the sensitivity of detection of a double-stranded DNA template by real-time PCR from a dilution series of *TNF* DNA template. The sensitivity was linear between 3 and 10,000 molecules of DNA template (**Figure 2a**). In order to examine the sensitivity of cDNA synthesis, we processed a purified *TNF* mRNA standard of known abundance as if it were a cell sample. The detection was background limited at ∼30 input mRNA molecules and was linear over more than two orders of magnitude (**Figure 2b**). The overall efficiency of mRNA processing/reverse transcription was ∼54%, based on a comparison between detection of same number of input mRNA and DNA molecules in the same experiment. In our hands, the efficiency of the reverse transcription step of an mRNA standard without the cell processing steps (lysis, DNase-I treatment) was similar (**Figure S1**).

**Figure 3:**
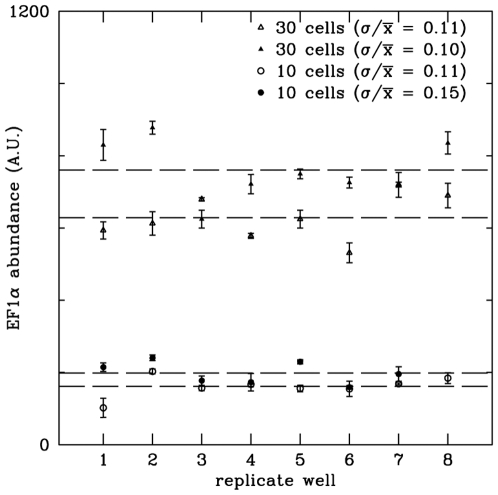
Precision of replicate real-time processing of single cells. Two sets of either 10 or 30 cell-samples were sorted by flow cytometry, lysed and then aliquoted into 8 aliquots. cDNA synthesis was performed independently for each aliquot and *EF1α* expression was measured by real-time PCR in triplicate on each aliquot. The replicates of each set of samples are shown (1.3-cell equivalents, open and filled triangles; 3.8-cell equivalents, open and filled circles). Based on this and similar experiments, we conservatively assign a fractional error of 15% to the cDNA synthesis step of our process.

We explored which of the mRNA processing steps was limiting the reaction efficiency. The use of gene-specific oligonucleotides (8 to 80 µM) to prime first strand cDNA synthesis improved the efficiency 2-3-fold compared to the use of oligo-dT priming (**Figure S2**). Using the same primer for both reverse transcription and subsequent real-time PCR was as efficient as a nested primer approach (data not shown). To determine whether our single-well procedure inhibited reverse transcription, we examined the length of the cDNA products after oligo-dT reverse transcription. No significant difference was observed between the abundance of a TNF mRNA standard measured by real-time PCR using either exon-1 or exon-4 primers/probes suggesting that long cDNAs (>1 kB) were being synthesized with oligo-dT priming (**Figure S3**). We also determined that moderate variations in annealing temperature (42–60°C) or reverse transcription time (50 min–2 hr) had no effect on the reverse transcription efficiency (data not shown). We also found that cellular RNAses were efficiently inhibited by the lysis solution because the presence of 10 cells in each well (6 µL) did not affect our ability to detect a spiked mRNA standard (**Figure S4**). The fractional error introduced by generating cDNA from cell samples was ∼15%, as demonstrated by processing replicates of the same cell lysate at the 1- or 3-cell level (**Figure 3**).

### Expression of innate immune genes in macrophages

**Figure 4:**
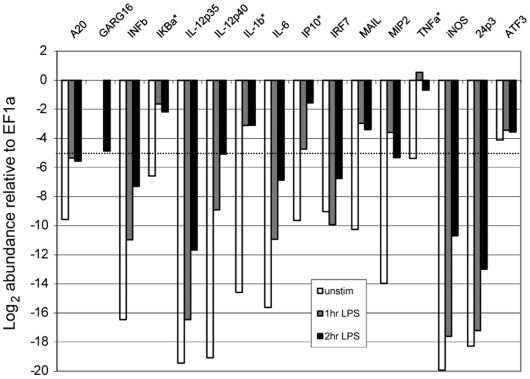
Relative abundance of immune genes in resting and activated macrophages. cDNA synthesis was performed from resting (open bar) and LPS-stimulated (1 hr, grey bar, or 2 hour, black bar) bone marrow macrophages (2.1×10^6^ cells). The indicated genes were detected by real-time PCR and their abundance (C_t_, mean of duplicates) is plotted relative to the *EF1α* signal (*EF1α* C_t_: Unstimulated = 18.54; LPS 1 Hour = 18.19; LPS 2 hours = 18.76). We estimate that we are able to detect gene expression within a C_t_ of 5 of *EF1α* signal (dotted line). An asterisk indicates the genes that were further investigated in single cells in the experiment shown in Figure 5.

As a guide to which innate immune genes we might expect to detect in single macrophages, we used real-time PCR to determine the relative expression of a panel of genes in a 2.1×10^6^-cell sample of primary murine bone marrow-derived macrophages (BMDM) under resting and stimulated conditions and then compared their expression to that of *EF1α* (**Figure 4**). The expression of *EF1α* did not change with stimulation (**See** **Figure 4** **legend**). Based on our sensitivity for detecting *EF1α*, we estimate that any gene with expression greater than ∼1/32 that of *EF1α* would be detectable in single-cell samples, and we selected a subset of these genes for further analysis.

### Coordinate expression of innate immune genes in single cells

In order to measure multiple genes from the same cell, we used pooled primers to amplify the genes of interest for 12 cycles (preamplification) prior to aliquoting the samples into separate wells for individual gene analysis (addition of probe). These additional steps had marginal effects on amplification efficiency across a range of input concentrations (**Figure S5**).

**Figure 5:**
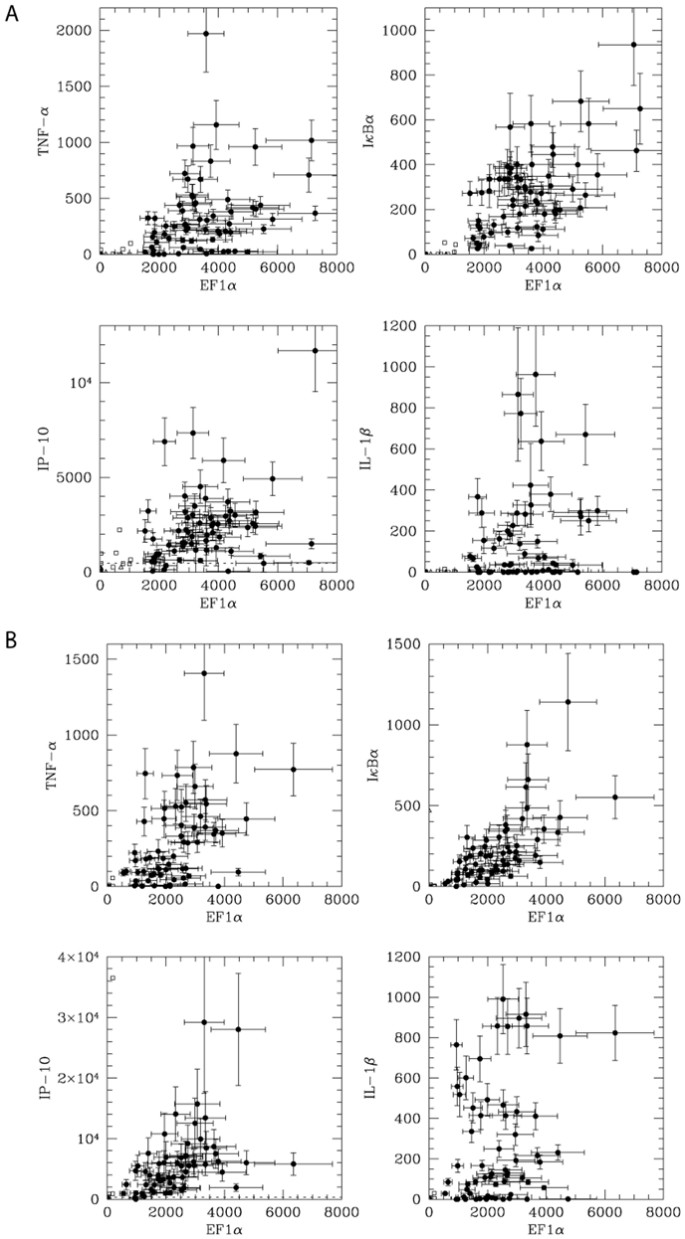
Simultaneous measurement of the expression of five genes in single macrophages. BMDMs were stimulated with A) poly I∶C or B) LPS for 2 hours and 84 single cells (together with 12 no-cell controls) from each experiment were sorted into a microtiter plate for cDNA synthesis. mRNA standards for each gene were used to calculate absolute expression values. In each panel, the abundance of *EF1α* is plotted on the X-axis, and the abundance of one of the other genes is shown on the Y-axis. This presentation permits the same cell to be identified in each panel, based on its level of expression of *EF1α* (position on X-axis). Negative controls include blank wells (no cell sorted, open triangles) and misses (open squares). A missed sample is defined as having an *EF1α* abundance <2x the highest value in “blank” wells. The detection limit for IP-10 (taken as 2x the highest value measured in “blank” wells) is indicated with a dashed line.

To examine the coordination of innate immune gene expression in macrophages, we measured 5 genes in each of 84 stimulated (poly I∶C) primary BMDM (together with 12 no-cell controls) (**Figure 5a**). We observed that the level of expression of these genes ranged over 2–3 orders of magnitude in these single-cell samples. Each gene had a different distribution and magnitude of expression in the population of cells. While IκBα was detected in all cells, only ∼60% of the cells produced measurable mRNA for *IL1β*. We did not observe significant coordinated expression between the panel of genes measured. While it is expected that individual cells will vary in their level of response, it is unexpected to find a lack of correlation between genes such as *TNF* and *IL1β*, which are activated by the same signal transduction pathway. A similar lack of coordinated expression was observed with LPS stimulation of BMDM macrophages (**Figure 5b**).

This cell-cell heterogeneity in gene expression occurs despite the apparent uniformity of the macrophage cell population, of which>99% were CD11b+ and F4/80+ as measured by flow cytometry. Additionally, the heterogeneity was not due to cell size variation, as strict gating on the FSC/SSC was used to limit the analysis to cells of uniform size/physical characteristics (<10% of total population) (**Figure S6**). Similar data were obtained using the cloned RAW 264.7 mouse macrophage cell line (data not shown), suggesting that cell heterogeneity is a general feature of macrophage biology.

**Figure 6:**
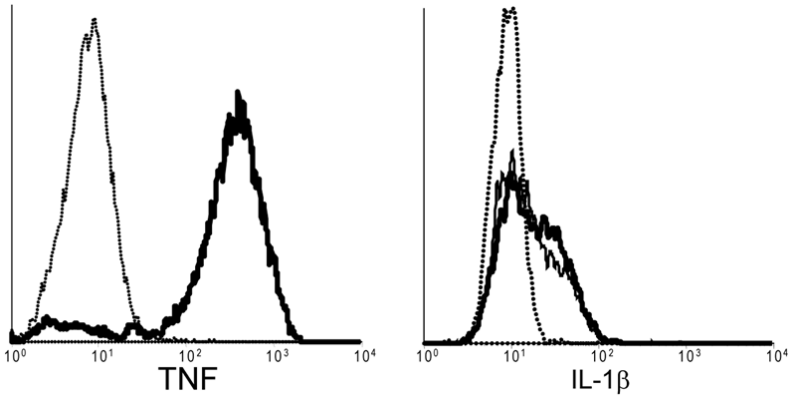
Distribution of cytokine protein expression in single cells. BMDMs were not stimulated (dotted line) or stimulated with for 4 hours in the presence of brefeldin A. The abundance of TNF and IL1β were detected by 2-color immunofluorescence and flow cytometry. The histogram shows the distribution of expression of the indicated cytokines in unstimulated cells (dotted line) and LPS (thick solid line) and poly I∶C (thin solid line) stimulated cells.

The cell-cell heterogeneity observed at the RNA level was also present at the protein level. After 4 hours of stimulation, TNF protein was detected in>90% of stimulated macrophages, while IL1β expression was only detected in 40% of the same cells (**Figures 6a, b**). The fraction of cells expressing IL1β protein was not greater than 40% at any time point measured between 0 and 8 hours (data not shown).

## Discussion

We measured the coordinated expression of innate immune genes in a population of macrophages with single cell resolution. During cell processing and measurement, we included RNA standards for each of 5 genes in order to identify the number of molecules of each gene expressed in each cell. While the level of heterogeneity across the cell population is expected based on single cell protein expression data, our data reveal an unexpected lack of coordination in the expression of immune genes. In individual cells, the levels of expression of pro-inflammatory genes such as *TNF* and *IL1β* were not correlated with each other or with the expression of the transcriptional inhibitor, *IκBα*. Similarly, expression of the cytokine IP-10 was not correlated with expression of *IκBα*.

These data suggest that the contributions of individual cells to the overall macrophage cytokine response vary widely. Furthermore, the expression of one cytokine in a given cell is not predictive of the expression of other cytokines within the same cell. Single-cell measurements provide the appropriate level of resolution and constraints for accurately defining the regulation of cellular behavior.

The sensitivity of detection with this approach varies by gene, but is typically limited by one of two factors. For some targets (e.g. *EF1α* and *IP-10*), the lower limit of detection is set by the background amplification seen in no-template controls (200–300 copies/reaction). Improved primer and probe design should significantly increase the sensitivity for these targets. For other targets (e.g. *TNF*, *IL1β*, and *IκBα*) negligible non-specific amplification is observed, and the detection sensitivity is set by the statistical fluctuations inherent in samples containing very small numbers of molecules. Improvements in cell lysis, mRNA extraction, and reverse transcription efficiency offer the potential to improve the detection sensitivity of these targets.

The key advantages of this single-cell mRNA detection method include the ability to measure the expression of 10's of genes from 100's of samples, to detect any target mRNA and to scale and automate. The procedure described here is not only applicable to single-cell samples, but can also be used for the multiplexed analysis of samples of limited availability, such as human tissue or blood. The single-well cDNA synthesis steps are fully compatible with alternative formats for real-time PCR analysis, such as the Fluidigm or Biotrove systems. Coupling this method to prior functional assessment of individual cells, such as by flow cytometry, imaging, or cell-based assays will dramatically increase the power of the technique to disentangle the subtleties of single-cell responses.

We anticipate that our single-cell analysis method will help resolve the complex cellular pathways underlying disease by overcoming the limitations of averaged cellular measurements, where responses that appear to be coordinated based on co-expression at the population level may in fact be unrelated at the single-cell level.

## Materials and Methods

### DNA standards

Double-stranded DNA standards for *TNF* and *EF1α* were cloned from C57BL/6 cDNA into the pEF6/TOPO vector (Invitrogen) and then subfragments were generated by restriction digest, purified and quantified using a spectrophotometer (Eppendorf Biophotometer).

### RNA standards

Single-stranded poly-adenylated RNA standards were generated for *TNF*, *IP-10*, *IκBα*, *IL1β* and *EF1α* by *in vitro* transcription from the cloned DNA using T7 polymerase and were quantified by a Bioanalyzer (Agilent) and confirmed as a single species.

### Ethics Statement

The animal use protocol used in this study was reviewed and approved by the Institute for Systems Biology's Animal Care and Use Committee (IACUC). The mice were euthanized by CO_2_ asphyxiation to minimize pain and distress, consistent with the recommendations of the Panel on Euthanasia of the American Veterinary Medical Association.

### Cell culture

Bone marrow-derived macrophages (BMDMs) were isolated from femurs and tibias of C57BL/6 mice. Bone marrow was cultured in RPMI 1640 media supplemented with L-glutamine, 10% FCS and human recombinant M-CSF (50 ng/mL). After 5 days of culture, cells were plated and used for experiments the next day. RAW 264.7 cells were obtained from ATCC (TIB-71).

BMDMs were stained using PE-conjugated CD11b (BD/Pharmingen #553311 rat anti-mouse IgG2bk) and FITC-conjugated F4/80 (Caltag RM2901-3 rat anti-mouse clone A3-1 IgG2b) to confirm the purity of the macrophages in the population.

### Cell stimulation

RAW 264.7 cells or bone marrow-derived macrophages were pretreated with γ−interferon (20 u/mL) (PeproTech) for 24 hours prior to stimulation with poly I∶C (10 ug/ml) (Amersham) or LPS (30 ng/ml) (*Salmonella minnesota* R595, LIST Labs) for 1 or 2 hours, as indicated. Cells were washed and kept on ice until flow sorting into microtiter plates containing CellsDirect Lysis buffer.

### Flow sorting

Cells or no-cell controls were sorted directly into PCR-compatible microtiter plates using an Influx flow cytometer (Cytopeia, BD). Narrow gates were set around the forward- and side-scatter distributions as well as the pulse-width measurement to guard against inadvertently sorting multiple cells. All sorting was performed with coincidence rejection enabled and the sort rate was maintained below 1000 events/s.

### Cell processing and cDNA synthesis

Cell samples were sorted directly into a 6 µl volume (5 µl Resuspension Buffer, 0.5 µl Lysis Enhancer, 0.5 µl RNase out, Invitrogen CellsDirect kit). We used half the reaction volume recommended by the manufacturer. All heating steps were performed in a PCR thermocycler (Applied Biosystems 7900). After sorting, each plate was immediately sealed and heated to 75°C for 10 min and either carried forward or frozen at −80°C. Each sample was incubated at room temperature for 10 min with 2.5 µl DNase I and 0.8 µl DNase I buffer to remove genomic DNA. We found that this step was dispensable and we omitted it for single or few (<10 cell) samples where contamination by genomic DNA was not a significant issue. When the DNase step was used, 0.6 µl of 25 mM EDTA was added to the sample, and each plate was then heated to 70°C for 10 min to inactivate the enzyme. cDNA was reverse transcribed using primers specific for each gene of interest in the sample (1 µl primer at 20 µM, 0.5 µl 10 nM dNTPs, 3 µl RT buffer, 0.5 µl RNaseOut, 0.5 µl SuperScript III RT, 0.5 µl DTT) (50°C 50 min, 85°C 5 min). The starting RNA was removed by adding 0.5 µl RNaseH (2 U/µl) and heating at 37°C for 20 min. Samples were subsequently stored at −20°C. We also prepared RNA from bulk macrophage samples using Trizol (Gibco) and performed reverse transcription using Superscript III (Invitrogen) with random hexamers. We used RNase-free solutions, and sterile, disposable labware, for all mRNA processing steps.

### Real-time PCR

cDNA was either measured directly or subjected to 12 cycles of pre-amplification with primers specific for each gene of interest prior to aliquoting for individual measurements (addition of probe) by real-time PCR. For direct measurement, we used 2 µl of cDNA sample, 10 µL 2X Fast Master Mix (ABI), 2 µL primer/probe (primer at 9 µM, probe at 2 µM) and 6 µL molecular grade water (Gibco). Standard cycling parameters were used. Whenever possible, samples and standards were analyzed on the same 384-well plate.

### Pre-amplification

Each cDNA synthesis reaction (16.4 µl) was combined with 5 µl 10X Master Mix (Applied Biosystems), 1 µl dNTP at 10 mM, 2.5 µl of each gene-specific primer pair at 10 µM, 0.25 µl Taq polymerase and brought to a final volume of 50 µl with molecular-grade water. Thermocyling was performed as follows: 94°C 3 min, 12 cycles of (94°C, 30 s; 50°C 30 s; 72°C, 45 s), 72°C 10 min. Real-time PCR was performed (7900HT, Applied Biosystems) on 2 µl aliquots of the pre-amplified reaction. Using this scheme, eight different transcripts can be measured in triplicate. For presentation, the Ct values (log_2_) were converted either to a log_10_ or linear scale.

Real-time data were analyzed using SDS 2.2.2 software (Applied Biosystems). Data were filtered by rejecting samples with a failed *EF1α* measurement, an abundance <2x the value in control (no cell) wells, or a real-time PCR amplification efficiency less than 1.7 (calculated by LinRegPCR[18]).

### Primers and probes

All genes were measured by qPCR with FAM/TAMRA-TaqMan reaction using primers and probes purchased from IDT (**Table S1**). We designed primers and probes using Primer Express software (Applied Biosystems) and confirmed their ability to detect a DNA standard for each gene, which was derived from mouse macrophage cDNA (RAW 264.7 and BMDMs).

### Intracellular cytokine staining

TNF and IL1*β* were detected by intracellular cytokine staining after 4 hours of stimulation with LPS in the presence of the protein secretion inhibitor, brefeldin A, as previously described [11]. TNF was detected using PE-conjugated antibody (554419, Pharmingen) and IL-1*β* was detected using a primary goat antibody (AF-401, R&D Systems) and a secondary FITC-conjugated anti-goat antibody (Jackson ImmunoResearch). Graphs were generated using WinMDI (Scripps).

## Supporting Information

Figure S1 Efficiency of cDNA synthesis. The fractional efficiency of cDNA synthesis (Ct abundance of mRNA vs. DNA) for TNF (solid square) and CXCL2 (solid triangle) was calculated using mRNA and DNA standards, for a range of input copy numbers expected for single macrophages (X-axis). Error bars represent the mean and SEM for six cDNA synthesis replicates.
(0.07 MB TIF)

Figure S2 Optimization of conditions for reverse transcription. Using a TNF mRNA standard, we performed reverse transcription using oligo dT (20mer), random hexamers or a gene-specific primer(R) and compared the yield of cDNA to the yield from a DNA standard. The gene-specific primer generated more cDNA than both random hexamers and oligo dT, across a range of input copy number. The concentration (1.5–5 uM) of reverse primer had no effect on yield.
(0.09 MB TIF)

Figure S3 Reverse transcriptase is processive to yield long cDNA. products. Macrophages were stimulated with LPS and different numbers of cells (1-, 3-, 10-, 30-, 100-cell samples) were sorted by flow cytometry into wells of a microtiter plate. After cDNA synthesis primed with oligo dT, the abundance of product (Ct) was detected by real-time PCR using primers/probe targeting sequences in exon 1 or exon 4 of the TNF gene (open circles, dashed line). The exon 4 probe had a 2-fold increased sensitivity over the exon 1 probe, which was consistent across samples of different abundance. This difference in probe sensitivity was not due to differences in reverse transcription, since it also was observed using a TNF DNA standard (10, 100, 1000, 10,000 copies) as the template (closed circles, solid line). We conclude that reverse transcription was not a limiting factor in the detection of TNF mRNA abundance by exon 4 or exon 1 primers/probe.
(0.41 MB TIF)

Figure S4 Effective inhibition of cellular RNases. cDNA synthesis and real-time PCR were performed on a dilution series of a TNF mRNA standard, either in the presence or absence of 10 unstimulated macrophages (distributed by flow cytometry). The TNF cDNA abundance (Ct, Y-axis) was similar whether macrophages were present or not during cDNA synthesis, indicating that cellular RNases were not degrading/inhibiting cDNA synthesis of the spiked TNF mRNA standard.
(0.09 MB TIF)

Figure S5 Efficiency of pre-amplification procedure. A dilution series was prepared containing pooled DNA templates for EF1alpha, TNF, IL-6, IkappaBalpha and IL-1alpha, and was pre-amplified by PCR for 12 cycles, before aliquoting into separate wells for individual gene analysis by real-time PCR. We compared the yield to that obtained from samples that were not preamplified. When corrected for sample volume (2 ul of a 50 ul reaction was measured for the pre-amplified samples) the measured differences in the mean Ct values for the same input copy number (EF1alpha, deltaCt = 7.56+/−0.08; IL-1beta, deltaCt = 7.0+/−0.1) were reasonably consistent with expectation for 12 cycles of amplification (deltaCt = 7.4) given typical pipetting accuracy. Data for EF1alpha and IL-1beta(which is representative of the results for the other genes) are shown. Error bars represent the mean and standard deviation for three replicate measurements of each sample by real-time PCR. For each gene, the doubling efficiency (epsilon), which was estimated by the slope of the dilution series, was similar for pre-amplified and non-pre-amplified samples.
(0.12 MB TIF)

Figure S6 Uniformity of bone marrow-derived macrophages demonstrated by co-expression of surface markers. After 5 days of culture, BMDMs were stained using A) isotype control antibodies, B) FITC-conjugated anti-CD11b antibody, C) PE-conjugated anti-F4/80 antibody and D) both anti-F4/80 and anti-CD11b antibodies. Samples in B and C were used to define gates. Essentially all the cells in the population (D) were dual positive for both macrophage markers, indicating that the measured heterogeneity in gene/protein expression in our experiments was not due to contamination by non-macrophage cells.
(1.35 MB TIF)

Table S1 Real-time Primer and Probe Sequences. Sequences for primers and probes used for Real-Time PCR
(0.06 MB DOC)
