# Supplementary figures and images for: Resolving Cell Population Heterogeneity: Real-Time PCR for Simultaneous Multiplexed Gene Detection in Multiple Single-Cell Samples
Source: PLoS One. 2009 Jul 27;4(7):e6326. doi: 10.1371/journal.pone.0006326 (PMC2711328; doi:10.1371/journal.pone.0006326)

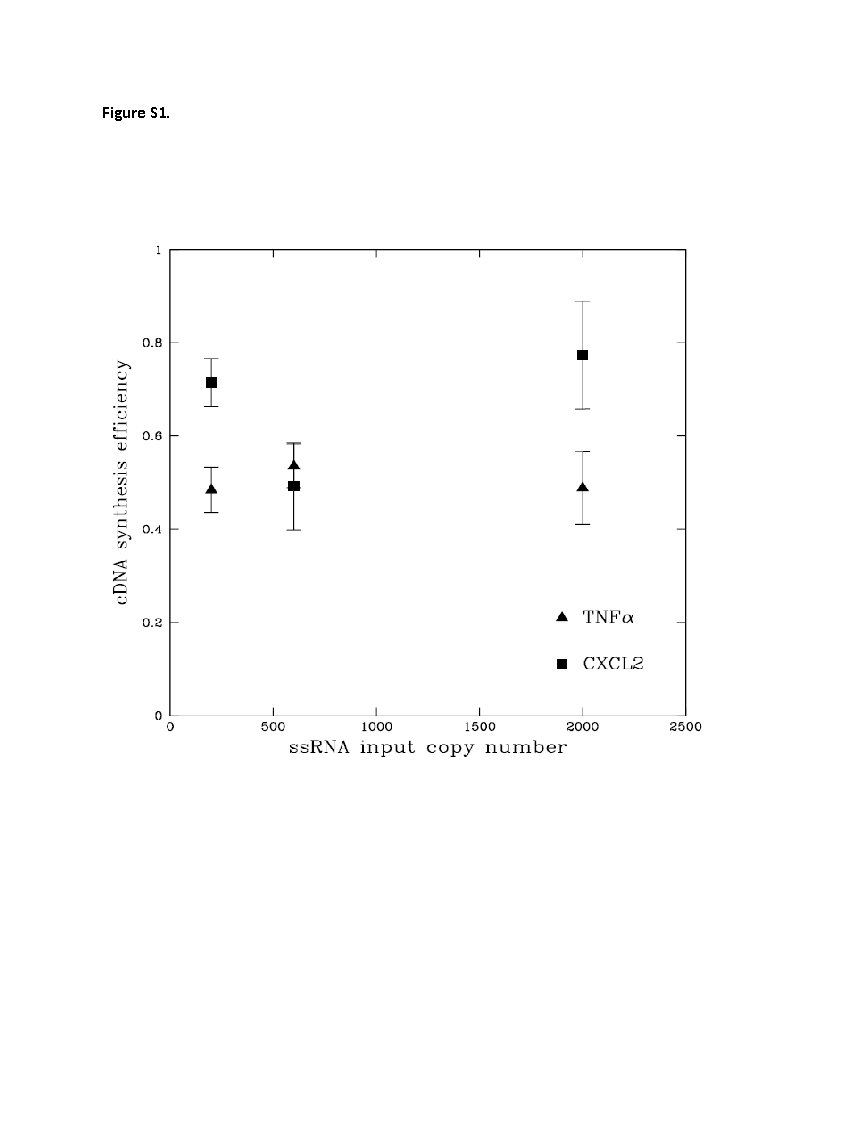

Supplement: Figure S1 — Efficiency of cDNA synthesis. The fractional efficiency of cDNA synthesis (Ct abundance of mRNA vs. DNA) for TNF (solid square) and CXCL2 (solid triangle) was calculated using mRNA and DNA standards, for a range of input copy numbers expected for single macrophages (X-axis). Error bars represent the mean and SEM for six cDNA synthesis replicates. (0.07 MB TIF) [file pone.0006326.s001.tif]

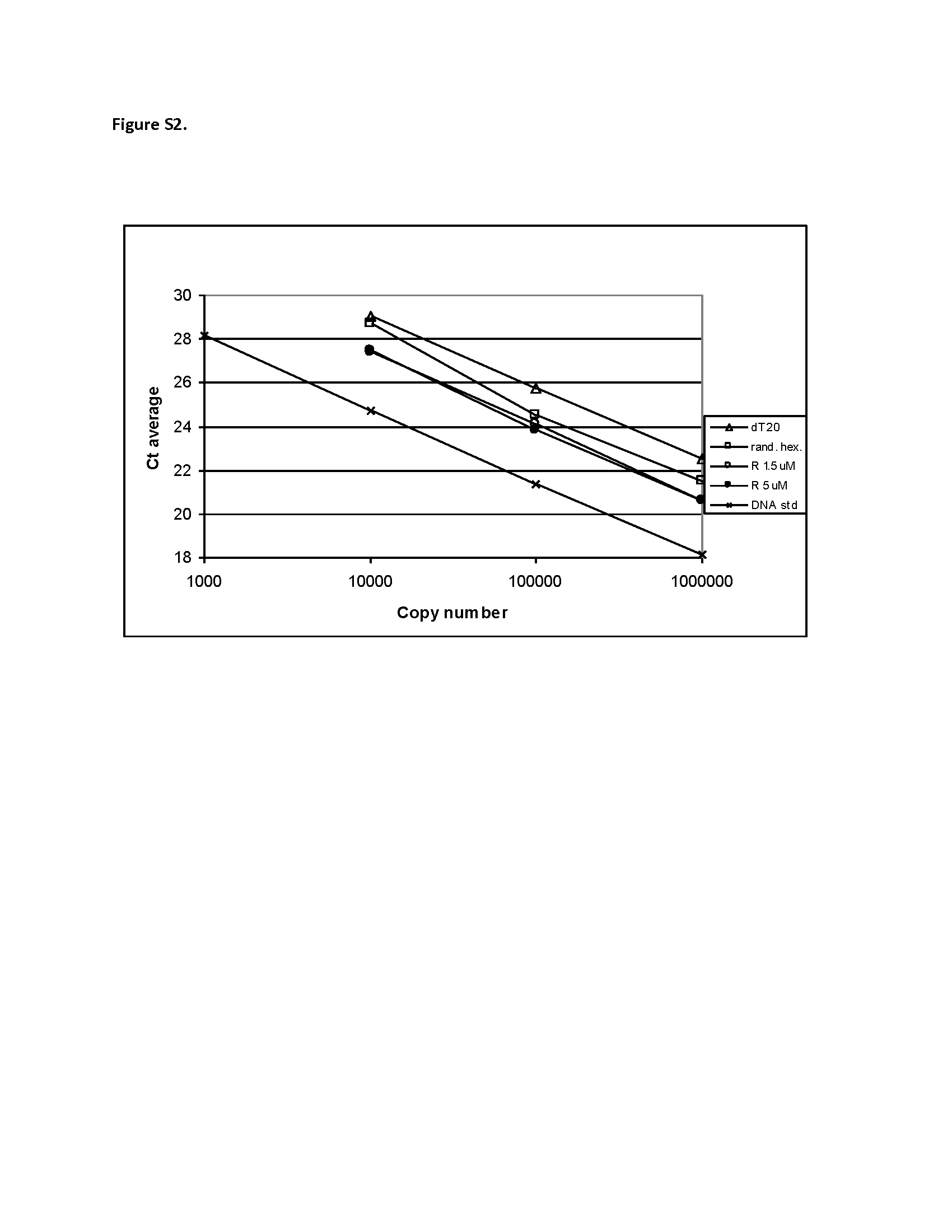

Supplement: Figure S2 — Optimization of conditions for reverse transcription. Using a TNF mRNA standard, we performed reverse transcription using oligo dT (20mer), random hexamers or a gene-specific primer(R) and compared the yield of cDNA to the yield from a DNA standard. The gene-specific primer generated more cDNA than both random hexamers and oligo dT, across a range of input copy number. The concentration (1.5–5 uM) of reverse primer had no effect on yield. (0.09 MB TIF) [file pone.0006326.s002.tif]

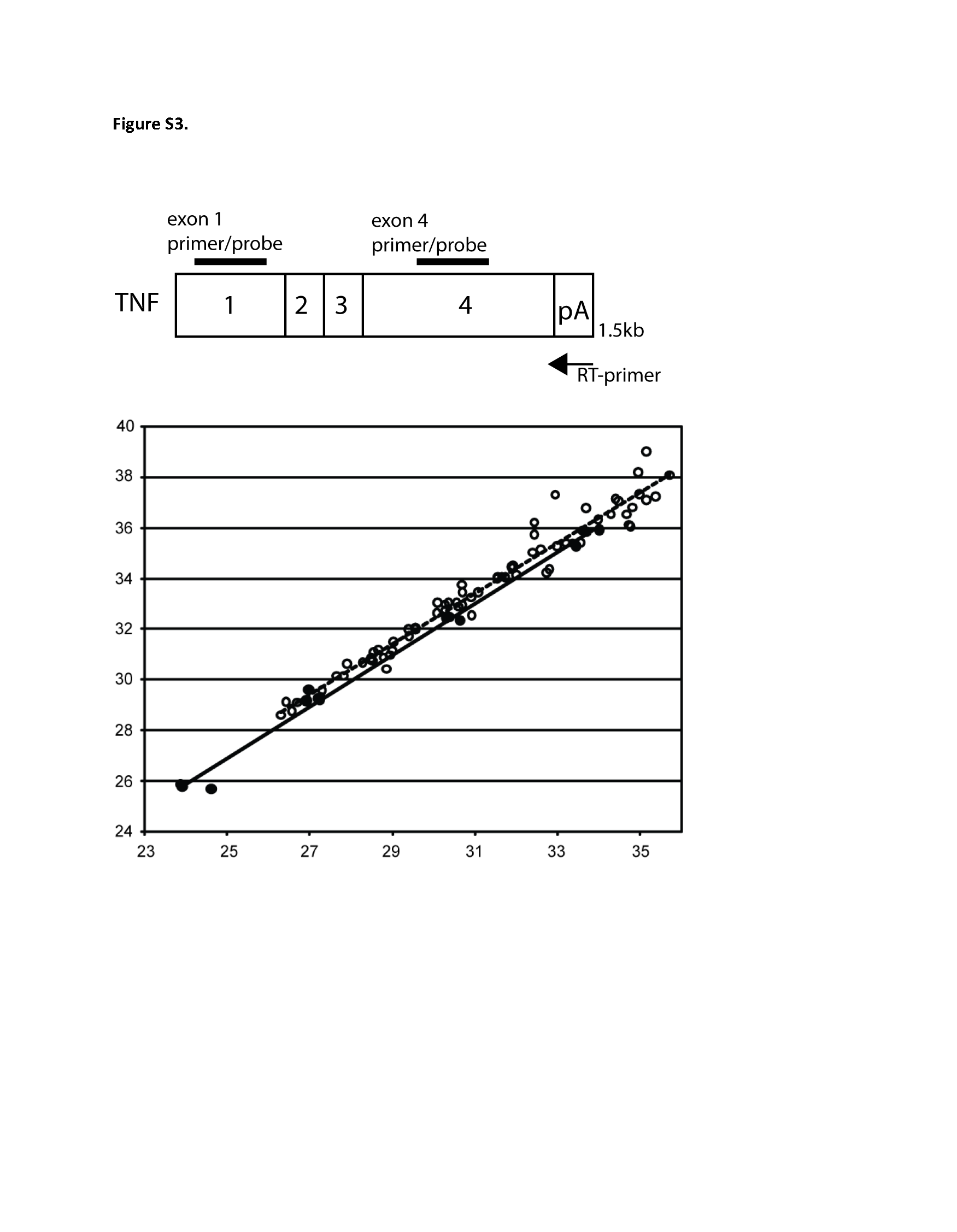

Supplement: Figure S3 — Reverse transcriptase is processive to yield long cDNA. products. Macrophages were stimulated with LPS and different numbers of cells (1-, 3-, 10-, 30-, 100-cell samples) were sorted by flow cytometry into wells of a microtiter plate. After cDNA synthesis primed with oligo dT, the abundance of product (Ct) was detected by real-time PCR using primers/probe targeting sequences in exon 1 or exon 4 of the TNF gene (open circles, dashed line). The exon 4 probe had a 2-fold increased sensitivity over the exon 1 probe, which was consistent across samples of different abundance. This difference in probe sensitivity was not due to differences in reverse transcription, since it also was observed using a TNF DNA standard (10, 100, 1000, 10,000 copies) as the template (closed circles, solid line). We conclude that reverse transcription was not a limiting factor in the detection of TNF mRNA abundance by exon 4 or exon 1 primers/probe. (0.41 MB TIF) [file pone.0006326.s003.tif]

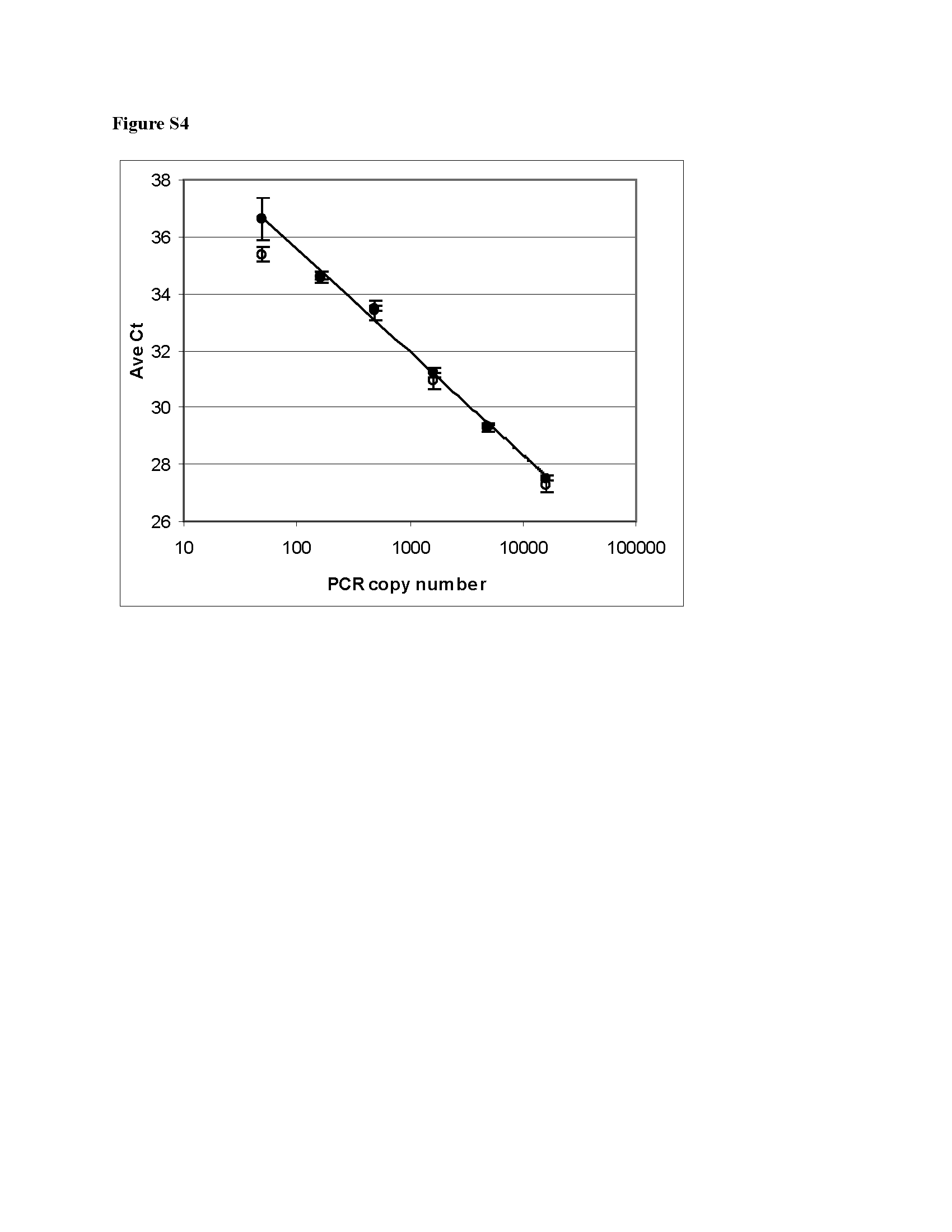

Supplement: Figure S4 — Effective inhibition of cellular RNases. cDNA synthesis and real-time PCR were performed on a dilution series of a TNF mRNA standard, either in the presence or absence of 10 unstimulated macrophages (distributed by flow cytometry). The TNF cDNA abundance (Ct, Y-axis) was similar whether macrophages were present or not during cDNA synthesis, indicating that cellular RNases were not degrading/inhibiting cDNA synthesis of the spiked TNF mRNA standard. (0.09 MB TIF) [file pone.0006326.s004.tif]

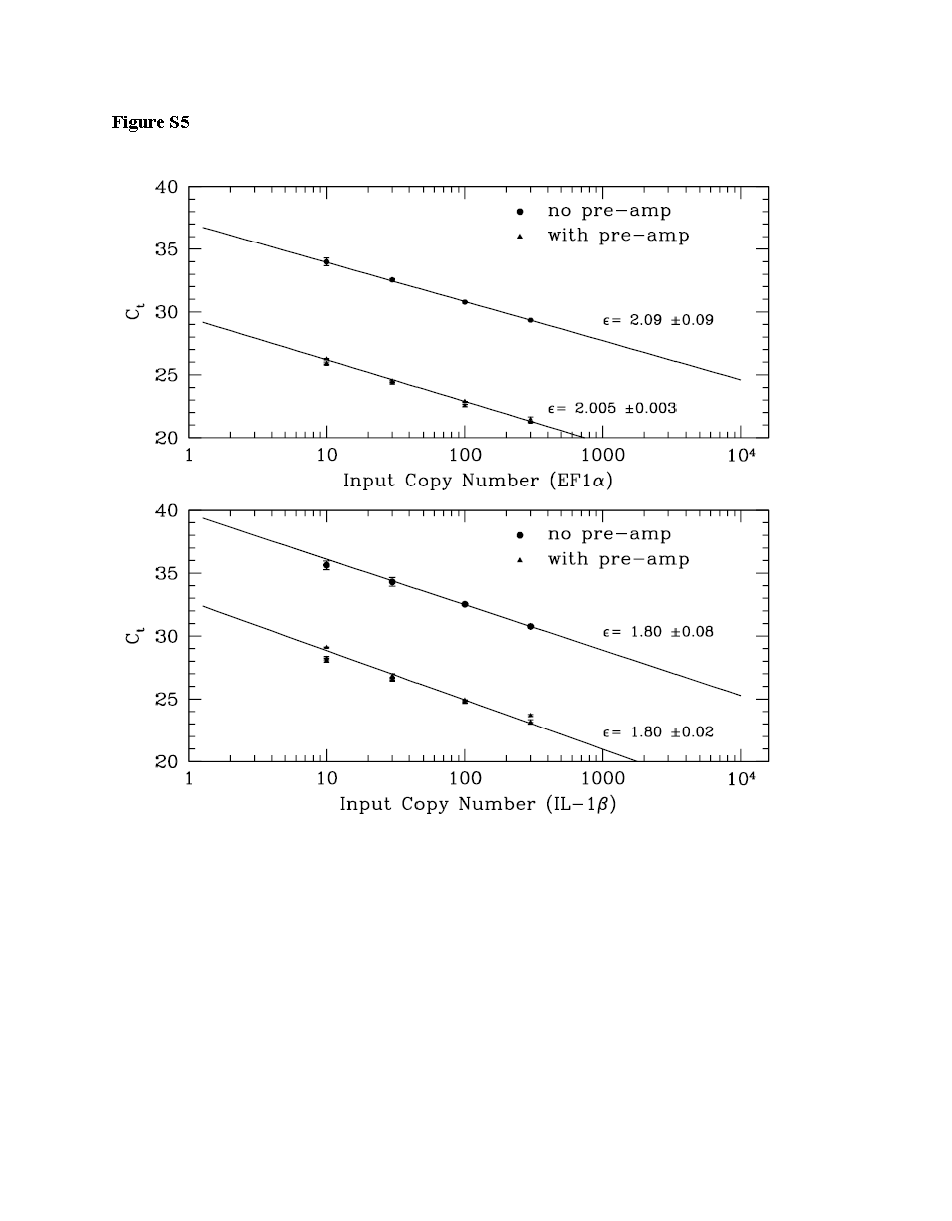

Supplement: Figure S5 — Efficiency of pre-amplification procedure. A dilution series was prepared containing pooled DNA templates for EF1alpha, TNF, IL-6, IkappaBalpha and IL-1alpha, and was pre-amplified by PCR for 12 cycles, before aliquoting into separate wells for individual gene analysis by real-time PCR. We compared the yield to that obtained from samples that were not preamplified. When corrected for sample volume (2 ul of a 50 ul reaction was measured for the pre-amplified samples) the measured differences in the mean Ct values for the same input copy number (EF1alpha, deltaCt = 7.56+/−0.08; IL-1beta, deltaCt = 7.0+/−0.1) were reasonably consistent with expectation for 12 cycles of amplification (deltaCt = 7.4) given typical pipetting accuracy. Data for EF1alpha and IL-1beta(which is representative of the results for the other genes) are shown. Error bars represent the mean and standard deviation for three replicate measurements of each sample by real-time PCR. For each gene, the doubling efficiency (epsilon), which was estimated by the slope of the dilution series, was similar for pre-amplified and non-pre-amplified samples. (0.12 MB TIF) [file pone.0006326.s005.tif]

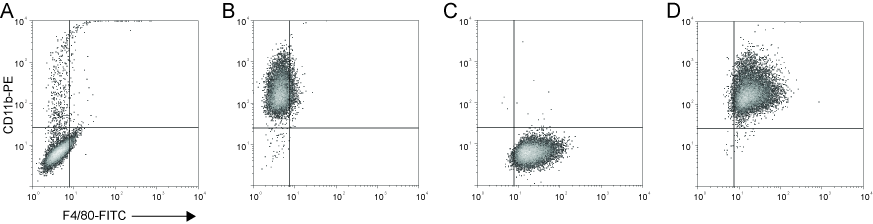

Supplement: Figure S6 — Uniformity of bone marrow-derived macrophages demonstrated by co-expression of surface markers. After 5 days of culture, BMDMs were stained using A) isotype control antibodies, B) FITC-conjugated anti-CD11b antibody, C) PE-conjugated anti-F4/80 antibody and D) both anti-F4/80 and anti-CD11b antibodies. Samples in B and C were used to define gates. Essentially all the cells in the population (D) were dual positive for both macrophage markers, indicating that the measured heterogeneity in gene/protein expression in our experiments was not due to contamination by non-macrophage cells. (1.35 MB TIF) [file pone.0006326.s006.tif]
