# Supplementary material for: Resolving Cell Population Heterogeneity: Real-Time PCR for Simultaneous Multiplexed Gene Detection in Multiple Single-Cell Samples
Source: PLoS One. 2009 Jul 27;4(7):e6326. doi: 10.1371/journal.pone.0006326 (PMC2711328; doi:10.1371/journal.pone.0006326)
Supplement: Table S1 — Real-time Primer and Probe Sequences. Sequences for primers and probes used for Real-Time PCR (0.06 MB DOC) [file pone.0006326.s007.doc]

**Supplementary Table 1: Real-time primer and probe sequences**

| Gene | Forward primer | Reverse primer | Probe |
| --- | --- | --- | --- |
| EF1a | GCAAAAACGACCCACCAATG | GGCCTGGATGGTTCAGGATA | CACCTGAGC AGTGAAGCCAG |
| TNF exon 1 | TCCAGGCGGTGCCTATGT | CACCCCGAAGTTCAGTAGACAG | TCAGCCTCTTCTCATTCCTGCTTGTGG |
| TNF exon 4 | CCAGGTTCTCTTCAAGGGACAA | CACACCGTCAGCCGATTTG | CTGCCCCGACTACGTGCTCCTCAC |
| CXCL2 | AGCCCCCCTGGTTCAGAA | GCTCCTCCTTTCCAGGTCAGT | TCATCCAAAAGATACTGAACAAAGGCAAGGC |
| IL1b | TCAGGCAGGCAGTATCACTCA | GGAAGGTCCACGGGAAAGAC | CTGTGGAGAAGCTGTGGCAGCTACCTG |
| IkBa | TTGGTGACTTTGGGTGCTGAT | GAGCGAAACCAGGTCAGGATT | AGCCCTGCAATGGCCGGACA |
| IP10 | GACGGTCCGCTGCAACTG | GCTTCCCTATGGCCCTCA TT | TCACTGGCCCGTCATCGATATGG |
| IL6 | CTGCAAGAGACTTCCATCCAGTT | AAGTAGGGAAGGCCGTGGTT | Biosearch Technologies, Ref# DNA16081A1 |
| Rantes | GGGAAAATCGGCATCTCCAT | TGCTGATTTCTTGGGTTTCGT | CGCTCCGACCGGCTCTCGAC |
| Mail | GATGGCCTGACTCCCCTACAT | CCGATTCCTCTGCAGTTCGT | ACCACCGCATTGTGAGCCACGA |
| A20 | AGCTGGCTGCATGTATTTTGG | CCAGGCACGGGACATTGT | CTCCAGAAAACAAGGGCTTTTGCA |
| Ifit1 (Garg16) | TGACATACCTGATTTGGAAGTGAGA | TGCATCCCCAATGGGTTCT | Biosearch Technologies, Ref# DNA17124B1 |
| IL-12p35 | GTCCAGCATGTGTCAATCACG | GCCAAACTGAGGTGGTTTAGGA | TACCTCCTCTTTTTGGCCACCCTTGC |
| IL-12p40 | GCTCAGGATCGCTATTACAATTCC | TCTTCCTTAATGTCTTCCACTTTTCTT | CTGCAGGGAACACATGCCCACTTG |
| IRF7 | TGGCAAGAGAAAATGCTGGG | GAGGTCCCCGGCATCACTA | TCCAAACCCCAAGCCCTCTGCTTT |
| Nos2 (iNOS) | GGCAGCCTGTGAGACCTTTG | GCATTGGAAGTGAAGCGTTTC | Biosearch Technologies, Ref #DNA17124C1 |
| IFNb | TGGCTTCCATCATGAACAACA | GAGGAGGGCTGTGGTGGAGAA | Biosearch Technologies, Ref #DNA6124G1 |
| 24p3 (lipocalin) | ACAAGCAATACTTCAAAATTACCCTG | TGGCAAAGCGGGTGAAAC | AACCAAGGAGCTGTCCCCTGAACT |
| ATF3 | ATGATGCTTCAACATCCAGGC | GGCCTTCAGCTCAGCATTCAC | Applied Biosystems Mm00476032_m1 |
